# iNaturalist as a tool in the study of tropical molluscs

**DOI:** 10.1371/journal.pone.0268048

**Published:** 2022-05-05

**Authors:** Rafael Masson Rosa, Daniel Caracanhas Cavallari, Rodrigo Brincalepe Salvador

**Affiliations:** 1 Departamento de Biologia, Faculdade de Filosofia, Ciências e Letras de Ribeirão Preto, Universidade de São Paulo, Ribeirão Preto, São Paulo, Brazil; 2 Natural History Department, Museum of New Zealand Te Papa Tongarewa, Wellington, Wellington Region, New Zealand; National Zoological Park, UNITED STATES

## Abstract

Although terrestrial gastropods are remarkably diverse, our knowledge of them is still lacking, especially for species from the Global South. As such, new tools to help researchers collect data on these organisms are very welcome. With this in mind, we investigated Brazilian observations on iNaturalist to assess the feasibility of the data available on the platform as a basis for studies on the tropical terrestrial gastropod fauna. The observations on iNaturalist were filtered by country, Brazil, and higher taxa, namely Eupulmonata, Cyclophoroidea and Helicinoidea, yielding a sample of 4,983 observations. These observations were then reviewed in search of records of rare or little-known species, species found outside their previously known range, and interesting ecological interactions. Exotic species made up 35% to 39% of the sampled iNaturalist records. The most commonly observed species were *Lissachatina fulica* (Bowdich, 1822), *Bradybaena similaris* (Férussac, 1822), *Drymaeus papyraceus* (Mawe, 1823), *Drymaeus interpunctus* (E. von Martens, 1887), *Limacus flavus* (Linnaeus, 1758), *Meghimatium pictum* (Stoliczka, 1873), *Cornu aspersum* (O. F. Müller, 1774), *Vaginulus taunaisii* (Férussac, 1821), *Ovachlamys fulgens* (Gude, 1900), and *Bulimulus tenuissimus* (Férussac, 1832). In total, 166 observations were deemed of interest to our purposes (e.g., rare species, range extensions, ecological interactions), totalling 46 identified species and 16 observations identified at genus level. Among the selected observations, we found pictures of live specimens of species that were previously known only from their shells, such as *Megalobulimus pergranulatus* (Pilsbry, 1901), bringing to light their appearances in life. Two potentially new species belonging to the genera *Plekocheilus* Guilding, 1827 and *Megalobulimus* K. Miller, 1878 were revealed. Additionally, we found records of living individuals of two species that were previously presumed to be possibly extinct, *Leiostracus carnavalescus* Simone & Salvador, 2016, and *Gonyostomus egregius* (Pfeiffer, 1845). We take the opportunity to discuss individual records of interest, evaluate the quality of the data and possible improvements, as well the potential and implications of the use of the iNaturalist platform for research in Brazil and other tropical countries. While iNaturalist has its limitations, it holds great potential to help document biodiversity in the tropics.

## Introduction

Terrestrial gastropods are one of the most diverse groups of molluscs, with estimates placing the number of described species at c. 24,380 [1]. However, many, if not most, species of terrestrial gastropods are still poorly known. There are significant gaps in our knowledge about their taxonomy, as a significant number of species remains undescribed [2]. Even among described species, there is a distinct lack of knowledge regarding their distribution and biogeography, as well as basic biology, such as life histories, feeding habits and habitat preferences [2, 3]. Many species were described only through their shells; the living animals have never been seen or recorded and nothing is known about the soft parts, not even how they look when alive [4]. Most of the available information on these subjects is reliant on data from European species, stemming largely from studies carried out from the 1800s to the mid-1900s [3]. Unsurprisingly, the knowledge of terrestrial gastropods in the Global South, where the vast majority of biodiversity is located [5], is lagging [6–8].

Despite all the difficulties, there are new emerging tools that can help researchers gain additional information, particularly in places of difficult or restricted accessibility such as the tropics [9]. One such tool is iNaturalist, a community-driven free-access platform for sharing observations of animals, plants and other organisms, accessible through a dedicated website and mobile application. iNaturalist is starting to enter the radar of researchers worldwide, who have identified its potential for research and conservation [10].

In the past few years, researchers have started to integrate iNaturalist data in their studies, particularly regarding the presence and distribution of species. Examples include studies on butterflies [9], termites [11], bees [12], flies [13], and molluscs [14, 15], including terrestrial gastropods [16]. Some of those studies also include observations of rare species [12, 16] and even the discovery of new species [13]. Other studies have found iNaturalist useful in detecting and recording the distribution of exotic species [17–20]. However, data stemming from community science initiatives can only be used for research if they meet certain quality standards [21].

Given the scarce natural history knowledge about terrestrial gastropods, especially in the Global South, we investigated their records on iNaturalist to assess how useful the platform can be for informing and enriching studies on these animals. To that end, we used Brazil as a case study, given its location in the tropics, its status as the most biodiverse country in the world [22], and the major place (for good or ill) social media platforms have in its present culture [23], as well as our expertise with its molluscan fauna.

## Materials & methods

To compile a list of all observations of terrestrial gastropods on iNaturalist, we filtered our searches by country (Brazil) and by the different higher taxa that include land snails and slugs, namely: Eupulmonata, Cyclophoroidea and Helicinoidea. Searches were conducted on February 1st, 2022, and resulted in a total of 4,983 observations.

We revised the identifications of all observations on iNaturalist using current literature (e.g., [24, 25]; and more specific taxonomic publications when necessary), as well as comparative specimens from the following natural history collections: CMRP (Faculdade de Filosofia, Ciências e Letras de Ribeirão Preto, Ribeirão Preto, Brazil), MZSP (Museu de Zoologia da Universidade de São Paulo, São Paulo, Brazil), and NMNZ (Museum of New Zealand Te Papa Tongarewa, Wellington, New Zealand). By doing that, we avoid the issues of using the raw data from iNaturalist, as even those observations tagged by the platform as “Research Grade”—that is, dated and georeferenced observations of wild organisms that are identified to species level or lower by at least two-thirds of the identifiers—are sometimes incorrect [26] (see also the Discussion below). When the previous identifications on iNaturalist were found to be incorrect, they were updated via our user profiles (two of the authors, DCC and RBS, are Curators on iNaturalist).

However, the animals in many observations cannot be identified due to several reasons, including poor-quality photographs, awkward angles, and diagnostic characters not being visible [26] (see also Discussion below). In those cases, we have offered identifications to the best level possible, be it genus, family, or superfamily.

**Fig 1:**
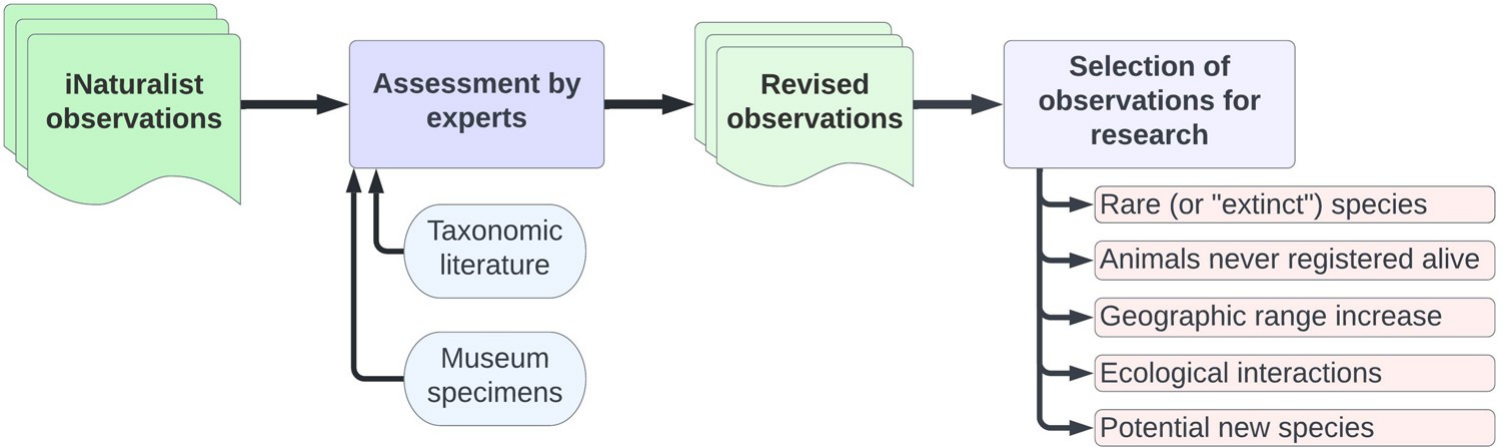
Workflow diagram. Diagram summarizing our workflow, starting with “raw” iNaturalist observations, through assessment of species identification, up to the selection of observations of interest.

After revising the identifications, we searched through the observations for entries of particular (and broad and/or more immediate) interest to the current research of terrestrial gastropods in Brazil. That includes rare species, species known only from shells, new distribution records, new records of exotic species, and potentially undescribed species. These entries were compiled and reviewed more thoroughly, being listed and discussed below. By “new distribution records” we are focused on large extensions of the species’ known range, such as records in a different state or biome. The geographic distribution of the species is known through works such as Simone [24] and Birckolz et al. [25], as well as more specific and recent publications (cited below when pertinent). A summary of the workflow can be seen in Fig 1.

## Results

**Fig 2:**
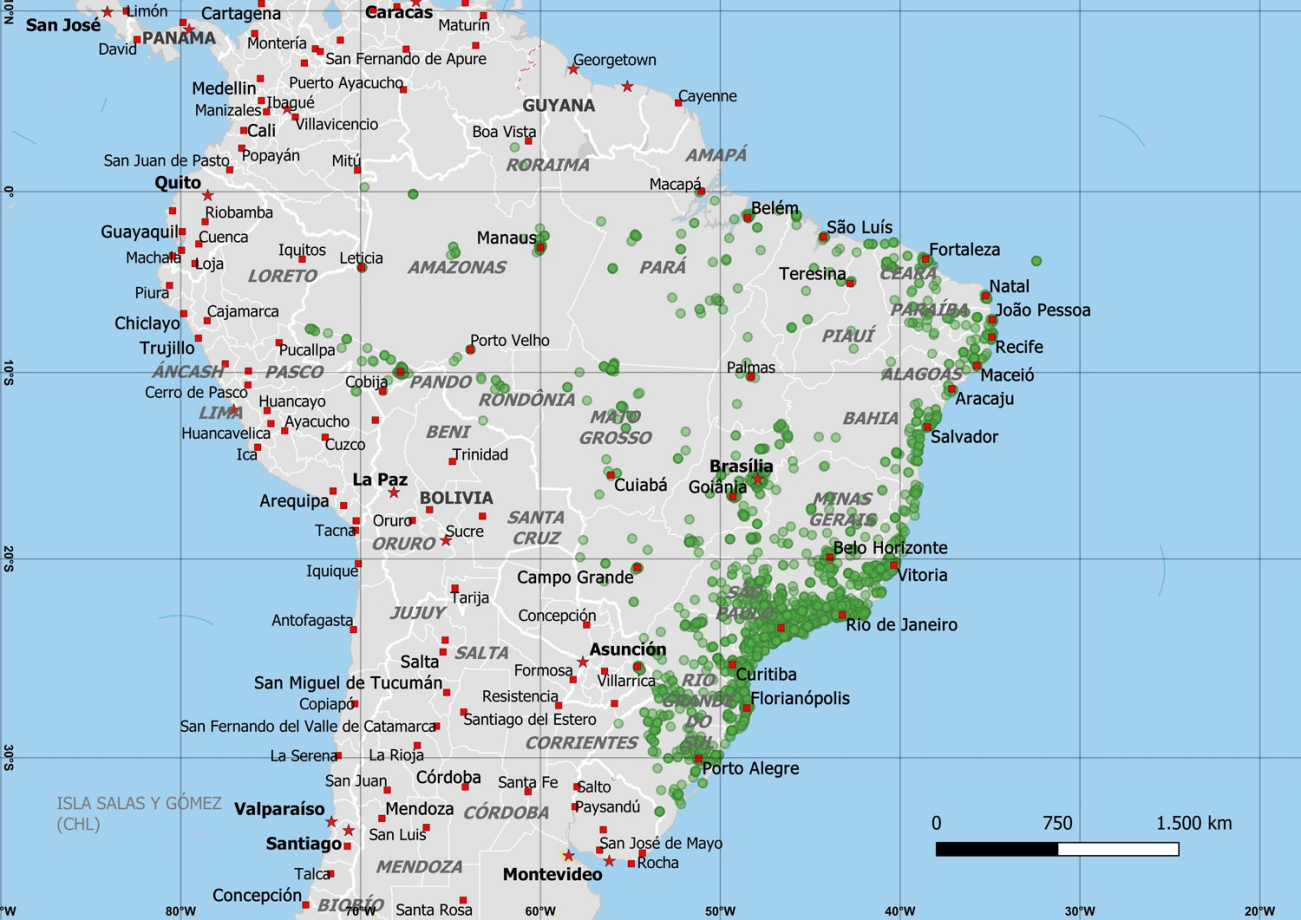
Geographical distribution of the observations of Eupulmonata on iNaturalist in Brazil. Eupulmonata is used as an example since it’s the gastropod taxon with the largest number of observations. In general, observations are mostly concentrated around large metropolitan areas. The map was created with QGIS software (https://QGIS.org) using map data from Natural Earth (https://naturalearthdata.com) and observation coordinates from iNaturalist.

As of February 1st, 2022, there were 4,983 observations of terrestrial gastropods in Brazil on iNaturalist (S1 File). iNaturalist launched in 2008, but some observations include photographs taken as early as 2002 (and 1982 in one case; S1 File). These were unevenly distributed across the different taxa, with 4,890 belonging to Eupulmonata, 13 to Cyclophoroidea, and 80 to Helicinoidea, which roughly corresponds to the diversity of each group in Brazil [8]. Likewise, the geographical distribution of the observations is uneven (Fig 2), being expectedly more numerous in metropolitan areas (see Discussion). Observations identified as exotic species make up 35% to 39% of these records, counting “Research Grade” observations only (1,732) or all observations (1,924). Since not all observations could be identified down to species level, the number of observed exotic species may be even higher.

There are just over 700 native species of terrestrial gastropods in Brazil [8]. After our revision, there are 185 identified species on iNaturalist (168 Eupulmonata, 6 Cyclophoroidea, and 11 Helicinoidea), although this number also includes exotic species and potential misidentifications (particularly of slugs) that our input on the platform could not correct. In addition, many observations are kept at the supraspecific level due to the impossibility of identification at the species level.

**Fig 3:**
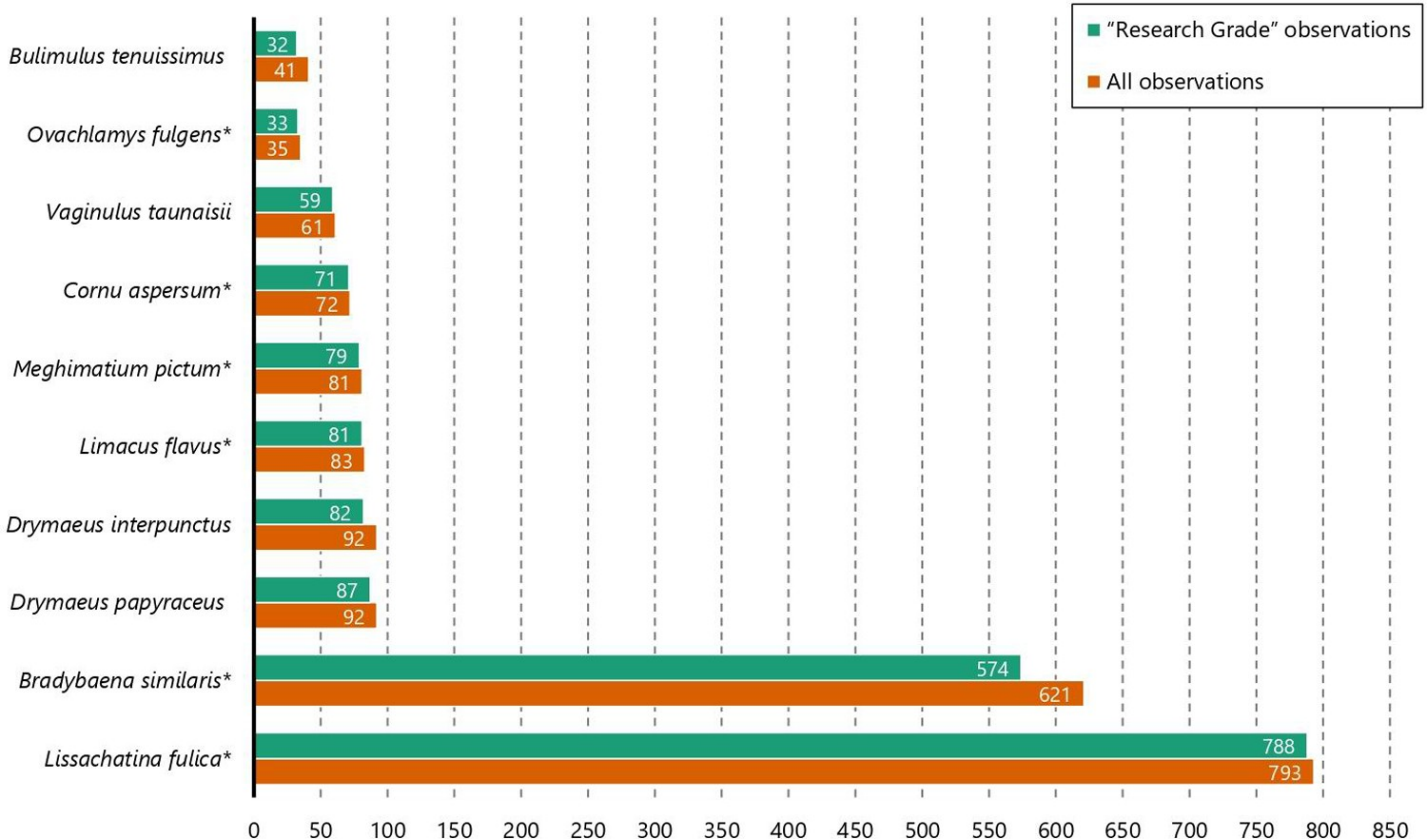
Breakdown listing of the top 10 terrestrial gastropods with the most observations published on iNaturalist in Brazil. “‘Research Grade’ observations” (after expert review) accounts for all observations listed as such on iNaturalist, while “all observations” contains all verifiable observations identified as each species. Exotic species are highlighted with an asterisk (*).

The 10 most commonly observed species are *Lissachatina fulica* (Bowdich, 1822), *Bradybaena similaris* (Férussac, 1822), *Drymaeus papyraceus* (Mawe, 1823), *Drymaeus interpunctus* (E. von Martens, 1887), *Limacus flavus* (Linnaeus, 1758), *Meghimatium pictum* (Stoliczka, 1873), *Cornu aspersum* (O. F. Müller, 1774), *Vaginulus taunaisii* (Férussac, 1821), *Ovachlamys fulgens* (Gude, 1900), and *Bulimulus tenuissimus* (Férussac, 1832). The records of the Veronicellidae slug *Sarasinula linguaeformis* (Semper, 1885) were excluded from this ranking (they would rank between *V*. *taunaisii* and *O*. *fulgens*) because a precise diagnosis from other species of *Sarasinula* is only feasible through penial anatomy or molecular data (e.g., [27, 28]). A breakdown of the ranking is shown in Fig 3. Notably, six out of the ten are exotic species; this matter will be more fully addressed in the ‘Biases’ section of the Discussion below).

In total, 166 observations were deemed of interest to molluscan research, amounting to c. 3.33% of the observations we analysed. Identification at the species level was only possible in 150 of the selected observations, totalling 48 identified species, while 16 observations were only identified at the genus level. The observations include new records of species that were generally considered rare or poorly known (i.e., species with few published records or studies), photographs of live specimens of species that were previously known only by shells, records of native species outside their previously known range, records of potentially undescribed species, and pictures of interesting ecological interactions. These records are listed in S2 File (summarised in Table 1), with some examples and highlights discussed in greater detail below. New records of exotic species outside their previously known distribution were not included in the table due to their large number, but the most important cases are also discussed below.

**Table 1:** Summary of records of interest (n = 166) for the present study compiled from iNaturalist, as of February 1st 2022.

Species			Recorded alive?	Nr of selected observations	Type of record
**HELICINOIDEA**					
	**Helicinidae**		** **	** **	
		*Helicina angulifera*	Yes	2	Range extension (to PE)
		*Helicina boettgeri*	Yes	1	Range extension (to RJ)
		*Helicina brasiliensis*	Yes	3	1st picture alive
		*Helicina rotundata*	Yes	1	Range extension (to PE)
		*Helicina wettsteini*	Yes	1	1st picture alive
**CYCLOPHOROIDEA**			** **	** **	
	**Diplommatinidae**		** **	** **	
		*Adelopoma paulistanum*	No	1	Rare/poorly-known
	**Neocyclotidae**				
		*Aperostoma amazonense*	Yes	1	1st picture alive; Range extension (to MT)
		*Aperostoma inca*	Yes	1	Range extension (to MS)
		*Aperostoma merrilli*	No	1	Range extension (to BA, GO)
		*Neocyclotus prominulus*	Yes	2	Rare/poorly-known
**EUPULMONATA**					
	**Achatinidae**				
		*Obeliscus boitata*	No	1	Rare/poorly-known
		*Synapterpes* sp.	No	1	Rare/poorly-known
	**Amphibulimidae**				
		*Plekocheilus* sp.	Yes	3	Potential new species
	**Bulimulidae**				
		*Anctus* sp.	?	1	Rare/poorly-known
		*Auris chrysostoma*	Yes	1	1st picture alive
		*Cochlorina* sp.	No	1	Predation record (prey)
		*Cochlorina uranops*	No	1	Rare/poorly-known
		*Drymaeus acervatus*	Yes	7	Rare/poorly-known
		*Drymaeus branneri*	Yes	1	1st picture alive; Range extension (to AC)
		*Drymaeus currais*	Yes	1	Rare/poorly-known
		*Drymaeus flexilabris*	Yes	4	Rare/poorly-known
		*Drymaeus gereti*	Yes	11	Rare/poorly-known
		*Drymaeus germaini*	Yes	1	Rare/poorly-known
		*Drymaeus magus*	Yes	5	Rare/poorly-known
		*Drymaeus poecilus*	?	2	Range extension (to GO)
		*Drymaeus rufolineatus*	Yes	16	Rare/poorly-known
		*Drymaeus semistriatus*	Yes	15	Rare/poorly-known
		*Drymaeus suprapunctatus*	Yes	3	1st picture alive
		*Pseudoxychona spiritualis*	Yes	1	Rare/poorly-known
	**Odontostomidae**				
		*Burringtonia* sp.	Yes	1	Rare/poorly-known
		*Burringtonia exesa*	Yes	2	Rare/poorly-known
		*Burringtonia labrosa*	?	1	Rare/poorly-known
		*Burringtonia pantagruelina*	?	1	Rare/poorly-known
		*Cyclodontina cylindrica*	No	1	Range extension (to GO)
		*Cyclodontina gemellata*	No	1	Rare/poorly-known
		*Cyclodontina tudiculata*	Yes	1	Rare/poorly-known
		*Moricandia angulata*	Yes	1	1st picture alive; Range extension (to RJ)
	**Orthalicidae**				
		*Orthalicus phlogerus*	Yes	2	1st picture alive
	**Scolodontidae**				
		*Scolodonta* sp.	Yes	2	Predation record (predator)
	**Simpulopsidae**				
		*Leiostracus carnavalescus*	Yes	3	Presumed extinct
		*Leiostracus demerarensis*	?	1	Range extension (to MT)
		*Leiostracus vimineus*	Yes	5	1st picture alive; Range extension (to PE)
		*Leiostracus vittatus*	Yes	2	Rare/poorly-known
	**Solaropsidae**				
		*Solaropsis feisthameli*	Yes	3	1st picture alive; Range extension (to MG)
		*Solaropsis penthesileae*	Yes	1	1st picture alive
	**Streptaxidae**				
		*Rectartemon* sp.	Yes	1	Rare/poorly-known
		*Rectartemon piquetensis*	No	1	Rare/poorly-known
		*Streptartemon* sp.	Yes	1	Rare/poorly-known
	**Strophocheilidae**				
		*Anthinus multicolor*	Yes	9	1st picture alive; Range extension (to ES)
		*Anthinus turnix*	Yes	7	Rare/poorly-known
		*Gonyostomus* sp.	Yes	1	Rare/poorly-known
		*Gonyostomus egregius*	Yes	1	Presumed extinct
		*Megalobulimus* sp.	Yes	4	Potential new species
		*Megalobulimus albus*	Yes	5	1st picture alive
		*Megalobulimus pergranulatus*	Yes	9	1st picture alive; Range extension (to MG)
		*Megalobulimus valenciennesii*	Yes	4	1st picture alive
		*Speironepion iguapensis*	Yes	1	1st picture alive
		*Speironepion pilsbryi*	Yes	2	Rare/poorly-known

Families are listed alphabetically for ease of use. Range extension pertains to new states (range extension of exotic species was not included); status as “rare/poorly-known” was defined based on expert knowledge of the organisms and the literature. Abbreviations of Brazilian states: AC, Acre; BA, Bahia; ES, Espírito Santo; GO, Goiás; MG, Minas Gerais; MS, Mato Grosso do Sul; MT, Mato Grosso; PE, Pernambuco; RJ, Rio de Janeiro.

In the Discussion below we will refer to some particular observations on iNaturalist by their internal register number in the database instead of presenting the full hyperlink. Each number can simply be added to the end of the following command “https://www.inaturalist.org/observations/” to become a functioning URL for accessing each observation on the iNaturalist website.

## Discussion

### Records of interest

The most immediate and obvious value of the observations on iNaturalist is in determining species’ geographic distribution. However, some records can have further scientific importance (Table 1; S2 File). To begin with, some observations can extend said geographic distributions, confirming that the animal in question inhabits a different area and sometimes biome, which is particularly important in countries of continental proportions such as Brazil. For example, *Helicina boettgeri* Wagner, 1910, previously known only from Espírito Santo and Minas Gerais states [25], has a new record indicating its occurrence in Rio de Janeiro state as well (observation 63533488). Similarly, *Drymaeus poecilus* (d’Orbigny, 1835), known from Mato Grosso, Mato Grosso do Sul, São Paulo and more recently Tocantins [29], now has two records confirming its occurrence in Goiás (observations 103752447, 31618865).

**Fig 4:**
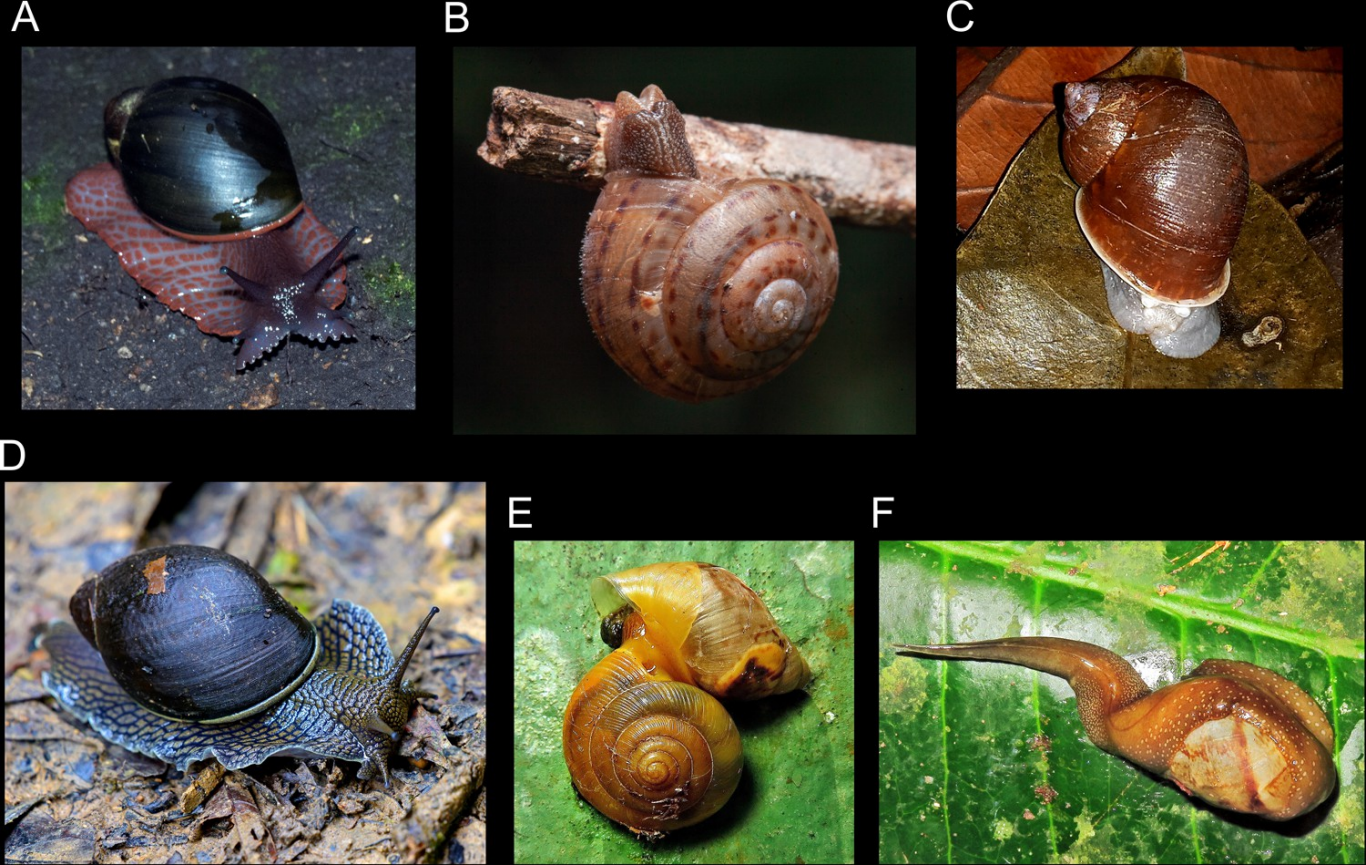
Examples of interesting observations on iNaturalist. Images were cropped to better display the animals. License is CC BY-NC 4.0 unless otherwise noted. A. *Megalobulimus pergranulatus* (observation 40731923, by marcosilva [Marco Silva], 26/Nov/2013). B. Penthesilea’s sundial snail *Solaropsis penthesileae* (observation 104759260, by projetomantis, 22/Oct/2021); © Leo Lanna / Projeto Mantis, used with permission. C. *Plekocheilus* sp. (observation 105830387, by gussoni [Carlos Otávio Gussoni], 27/Jan/2022). D. *Megalobulimus* sp. (observation 18383527, by rondon, Nov/2018). E. *Scolodonta* sp. preying on a Bulimulidae (observation 95894827, by rogerriodias [Rogerio Dias], 20/Sep/2021). F. *Cochlorina* sp. being preyed upon by the flatworm *Obama burmeisteri* (observation 69277257, by rogerriodias [Rogerio Dias], 07/Jan/2017).

In addition, many snail species are known only from dry shells, usually collected more than a century ago and now kept in museum collections. The photographs on iNaturalist often show the animals’ soft parts, so researchers and conservationists can know what the actual live animals look like. We identified a few such cases, such as *Drymaeus branneri* F. Baker, 1914 and *Megalobulimus albus* (Bland & Binney, 1872), but the most striking example is *Megalobulimus pergranulatus* (Pilsbry, 1901). This species had never been photographed alive before and the present observations have brought to light that this animal’s soft body has a distinct color (orange and blueish-gray; Fig 4A), which turned out to be a very important diagnostic character for taxonomy (José H. Fontenelle, pers. comm.). In a similar case, Penthesilea’s sundial snail *Solaropsis penthesileae* Salvador, 2021 has been recently described based on an ethanol-preserved specimen from a natural history collection, collected nearly 25 years ago [30]. A record of a live specimen of that species was submitted to iNaturalist in January 2022 (Fig 4B; observation 104759260).

A further–and unexpected–use of iNaturalist’s observation was to assess that a presumed extinct species [31], the harlequin snail *Leiostracus carnavalescus* Simone & Salvador, 2016, is thankfully alive and well (observations 59172104, 82429840, 103928060). Similarly, *Gonyostomus egregius* (Pfeiffer, 1845) has been considered possibly extinct in the recent past [32, 33], but an observation dating from 2009 shows what seems to be a living individual (observation 68705299), indicating that it was still extant, albeit rare, at the time of its first assessment and increases the likelihood of its continued existence today.

One of the most striking uses of iNaturalist observations, however, is in detecting potential new species, previously unknown to science. This has already been documented for organisms going from flies [13] to frogs [34]. Among the present records, we spotted two potentially new species belonging to the genera *Plekocheilus* Guilding, 1827 (Fig 4C; observations 35742586, 99302855, 105830387) and *Megalobulimus* K. Miller, 1878 (Fig 4D; observations 18383527, 68165703, 102418517, 104528656).

Photographs on iNaturalist can also capture important ecological information. For instance, we detected two records of *Scolodonta* sp. preying on other snails, apparently Orthalicoidea (Fig 4E; observations 95894827, 103817465). These records are of particular importance, given that the carnivorous habits of Scolodontidae have been called into question before [35]. We have also identified one record in which a *Cochlorina* sp. is being predated by a land planarian of the species *Obama burmeisteri* (Schultze & Müller, 1857) (Fig 4F; observation 69277257). The ecology of terrestrial flatworms is scarcely investigated worldwide, particularly in the neotropics where they are extremely diverse [36–38], so records of predation events (especially identifying the prey species) are always welcome.

Finally, iNaturalist can be extremely useful to keep tabs on exotic species, which brings us back to the topic of geographic distribution, only this time applied in a different context. iNaturalist not only keeps track of the location of each observation, but also when they took place, so it is possible to get an idea of how exotic species are spreading throughout the country. The distribution of exotic land snails and slugs in Brazil has never been particularly well documented, barring some exceptions like the giant African snail *Lissachatina fulica* (Bowdich, 1822), so there is ample opportunity for iNaturalist observations to help. For instance, the two-toned hunter snail *Gulella bicolor* (T. Hutton, 1834) was previously known only from Rio de Janeiro state in SE Brazil and Acre state in the westernmost Brazilian Amazon [39], but one iNaturalist record (observation 42475972) indicates this species presence in Pará state, a locality far removed from the previous occurrences.

While many exotic species, including *Gulella bicolor* above, have been present in Brazil for some time [24, 40, 41], two recently-detected species warrant further discussion. The Japanese jumping snail *Ovachlamys fulgens* (Gude, 1900) was first detected in São Paulo state [42] and later recorded from Rio de Janeiro to Santa Catarina states [43]. A record from late 2021 on iNaturalist extends this distribution to the north, to Espírito Santo state (observation 98824918), indicating a rapid spread of this species in the country. Likewise, *Macrochlamys indica* Godwin-Austen, 1883 has been detected in southern Brazil quite recently [44], but has iNaturalist records from the following additional states: São Paulo, Minas Gerais, Goiás, Mato Grosso, Acre and Amazonas. The fact that it is already present in the Amazon, far from the major urban centres, indicates that this species had been present in Brazil undetected for a long time.

### Quality of records

Taxonomic identifications on iNaturalist are recurrently problematic due to several factors ranging from the poor quality of photographs to the platform’s AI-based ID (identification) system [26]. Photographs are often awkwardly positioned, have low resolution, lack proper scaling, and do not highlight any diagnostic characters. Determining the identity of shelled gastropods usually requires multiple views of the shell (e.g., a view of the shell’s aperture is required to identify most Odontostomidae). Obtaining such images would require the photographer to handle the specimens, which is generally not an advisable course of action in most contexts and should not be expected of iNaturalist’s general public. Moreover, some groups cannot be identified at the species level without dissection, as diagnostic characters are often related to internal features of the reproductive system, which in our case is true for nearly all the Veronicellidae [27, 28].

Internal validation on iNaturalist requires that at least two users out of three endorse any given identification for an observation to achieve a so-called “research-grade”. This seemingly democratic system is hampered by a few problems, such as users validating misidentifications suggested by the platform’s AI (which typically suggests European or North American taxa, even for observations in Brazil or elsewhere). As the system ranks users based on the number of IDs, “clickers”, i.e., users who want to accumulate as many IDs as possible regardless of whether they are correct or not, make the problem even worse [26], even though this has not happened frequently in the observations analysed herein. Furthermore, when a mistaken ID is extensively validated by users, correcting the error becomes nearly impossible. This type of problem stems from the fact that the validations made by any user (be them experts or not) have the same weight.

In any case, problems with photographs could be solved or at least alleviated by simple measures. Providing quick guides could show users which structures or viewing angles help specialists to identify a particular taxon, considering the limitations of commonly used equipment (smartphones or less sophisticated digital cameras). These guides could be available to users on the spot: based on the first uploaded image, the AI identifies the greater group to which the specimen belongs (e.g., Class Gastropoda), prompting the user to take additional images. Simultaneously, general guides could be made available to the community at large, with detailed instructions depending on each taxonomic group.

Vandalism (i.e., clickers) is a bit harder to tackle, but these individuals seem to be a small fraction of the iNaturalist community and are often predictable. They could be identified and automatically flagged by AI or actively by Curators. Suffering from similar vandalism issues for a long time, Wikipedia has developed effective anti-vandalism mechanisms that could inspire future solutions for iNaturalist [45]. Nevertheless, as explained above, even if vandals are halted, the potential damage done to the identifications may be hard to revert. Assigning greater weight to identifications made by curators and specialists, as well as providing tools so that qualified users can restart the identification process are some paths towards a possible solution.

To be usable in research, community science datasets need to be of high quality [21]. So, validation by experts is essential to ascertain the accuracy of the identifications and the benefits of the increasing engagement of scientists on the platform have already been pointed out [26]. In this context, Brazilian reality imposes some difficulties, starting with the small number of experts, who are often too overloaded with activities to be able to interact with the platform. The contributions of experts from other Latin American countries and elsewhere were relevant to verify several observations analysed here, which also highlights potential benefits of the platform as a place for information exchange between professionals, networking and establishing collaborations.

In addition, if experts active on iNaturalist take the time to explain how a given species was identified, this can provide a valuable experience for community scientists. With time, community scientists will achieve a similar level to experts [21]; iNaturalist already counts with many of these “expert observers” [9], who are community scientists with ample knowledge of particular taxa.

### Biases

In addition to the aforementioned problems, there are also clear biases when it comes to which species are observed on iNaturalist. Most of the observations consist of species that are either large (e.g., *Megalobulimus* spp.), conspicuous (e.g., colourful *Drymaeus* spp.), abundant in or near urban areas (e.g., the exotic *Bradybaena similaris*), or a combination of these factors (e.g., the exotic *Lissachatina fulica*, which is by far the most observed mollusc in Brazil). In contrast, species that are smaller and less flashy often go unnoticed, as do those that are more commonly or exclusively found in areas with little human activity. Some snails also live on trees (e.g., some Orthalicoidea) or in the leaf litter in the ground (e.g., Punctoidea) and can go unnoticed as well, and some may even be too small for commonly used cameras to capture properly (e.g., some Pupilloidea).

On the bright side, this means that iNaturalist can be a great tool for monitoring exotic species since they are usually quite easy to spot and tend to live near human settlements. This has already been demonstrated in previous studies [17–20] and is reiterated here by the fact that 6 out of the 10 most observed species in Brazil are exotic (Fig 3). As we mentioned above, the data from iNaturalist was useful to provide new information on the poorly documented and quickly expanding distribution of *Ovachlamys fulgens* and *Macrochlamys indica* in Brazil. With the large influx of new observations every day, it seems likely that further surveys on iNaturalist could uncover new data and potentially help to identify newly-arrived exotic species.

### Potential uses of record

Information gathered from iNaturalist can be quite useful for several purposes. As discussed above, the most obvious information that can be collected from the site is new data on the distribution of observed species. This is potentially very useful, since this sort of data is essential to assess the conservation status and develop solid conservation plans for these animals, which are among the most threatened in the current biodiversity crisis [2, 33]. The records on iNaturalist also include those made in remote areas and private land, which are often not easily accessible to researchers [9]. In addition, filling the gaps in our knowledge about the distribution of these species is a necessary step to carry out further biogeographic (and sometimes taxonomic) research on them.

In some cases, observations can provide interesting anatomical and taxonomic information, especially for species that were initially described only from shells or other incomplete material. Some observations were able to bring forth new and unexpected information about the soft body morphology of some species, as was the case of *Megalobulimus pergranulatus*. Pictures of live specimens are always welcome in such cases, as some species of gastropods can look completely different when alive in contrast to dry shell material. Live observations can also provide data on a species’ ecology, especially when including records of ecological interactions with other organisms, such as predation or mating. Even regular pictures could offer clues about the species’ preferred microhabitat.

The data on iNaturalist can also be used to plan future collections, as records of a species will often be concentrated around a specific area. This can be very useful to collect new material for species that are poorly represented in scientific collections, but it also comes with its disadvantages. Namely, this data could also be used by amateur collectors and shell dealers, which could lead to the overcollection of species that are often already rare. Overcollection for non-scientific purposes is already one of the largest threats to the conservation of molluscs in the tropics [46–48] and access to detailed distributional data of these species can potentially aggravate this problem even further. To prevent this, iNaturalist has a system of taxon geoprivacy in which the location of an observation is automatically obscured and replaced with less specific information in cases where the observed species are considered globally or locally endangered [49]. This system is based on the conservation statuses added to the iNaturalist database and, while not perfect, it can certainly be very useful to avoid poaching of well-documented species such as large vertebrates and plants. However, there is a serious and very worrying lack of assessments on the conservation status of molluscs and invertebrates in general, particularly in the tropics [33, 50], which makes us unable to accurately determine which species should be considered endangered.

Lastly, the great potential that iNaturalist holds for documenting exotic species, both geographically and through time, is perhaps one of its most valuable uses. It could be used to inform countermeasures against these species’ invasions, especially by allowing the quick identification of occurrences and potentially locating invasions that are still in their early stages. If exotics are identified early on, it is more likely that they can be controlled and eradicated [51].

## Conclusions

Considering the gaps in our present knowledge, the difficulties of studying tropical invertebrates, and the reduced number of scientists [9], harnessing the power of the community is essential. iNaturalist, being an easy-to-use and fun app, fits the bill nicely. And when the data is curated and validated, it can help researchers in their study. In effect, it has already been helping in small ways, both in Brazil [16] and worldwide [12–15]. However, there is ample potential for more: the results from our survey of observations in Brazil have already brought to light new data. And we only considered singular records, so there is plenty of room for wider studies (e.g., distribution of native and exotic species) and more specific research questions.

The challenges faced by taxonomic research on gastropods in Brazil are numerous [8], and the current context of ongoing budget cuts and undermining of science in the country have done nothing but worsen the situation to a critical point [52–56]. In face of such a dire scenario, a free application that, by itself, encourages interest in biodiversity and science, certainly helps to reverse this dramatic situation, since popular interest is a necessary factor in promoting public policies. Moreover, if the scientific community sometimes fails in science outreach initiatives, apps like iNaturalist can help in filling the space left by this communication deficiency by encouraging people’s interest in biodiversity.

Our study focused on Brazil, but it serves as an example of the usefulness of iNaturalist that can be replicated elsewhere around the tropics. It is particularly helpful that most of the mega-diverse countries in Latin America, Africa, and Southeast Asia are also the most hyper-connected and social-media-prone in the world [57]. Still, documenting nature as a leisure activity has yet to take root in those countries, as most observations on iNaturalist come from countries of the Global North (and roughly half the observations are from the USA alone, as of February 1st 2022). For instance, New Zealand, a small country with a small population, has more iNaturalist observations than mega-diverse and hyper-populated India (as of February 1st 2022). India does not make the cut as one of the most connected countries worldwide given that pertains to the proportion of users, but it is way ahead in the sheer number of social media users [57], so there is potential there to be “diverted” to iNaturalist. In addition, the usage of iNaturalist by community scientists in Global South countries may also help in bringing back a bit of the power and the ability to conduct research to locals. Those countries, whose science is typically underfunded and lacks infrastructure, have been and still are a target for colonialist practices [58, 59].

Clearly, iNaturalist users alone will not be able to document biodiversity in all corners of the tropics anytime soon [9, 10], but it is already a start and, as shown above, it does produce important data for research. One obvious way of improving the platform is to increase the number of people contributing. Besides an obvious increase in advertising campaigns and the good old word of mouth, local programs and events can contribute towards this goal. BioBlitzes, for instance, despite typically being used for education and outreach, can also be applied for research purposes [60, 61]. If organised including the uploading of data on iNaturalist, the potential of these activities for research would be greater. Likewise, more focused research projects by scientists working in universities or museums can be organised around community science and iNaturalist to obtain quality data (e.g., [62]). Learned societies can also play a role in organizing projects, involving non-scientist members and non-members alike, that harness the power of iNaturalist (e.g., [63]). Finally, global programs and events are also a reality. Since 2016, iNaturalist has promoted the City Nature Challenge (https://citynaturechallenge.org/), in which people contribute with observations of urban and suburban areas in several cities around the world.

Now is a good moment to capitalise on people’s rekindled interest in nature found during the COVID-19 pandemic and the numerous lockdowns around the world [64]. If that impetus can be diverted to activities involving iNaturalist, an already useful biodiversity database can reach new levels of excellence and help enable research in the megadiverse regions of the world where it is most needed.

## Supporting information

S1 File Compilation of all observations of terrestrial gastropods on iNaturalist in Brazil as of February 1st, 2022. (XLSX)

S2 File Comprehensive list of all records considered of interest to this paper. (XLSX)
